# Mechanisms of FSH- and Amphiregulin-Induced MAP Kinase 3/1 Activation in Pig Cumulus-Oocyte Complexes During Maturation In Vitro

**DOI:** 10.3390/ijms20051179

**Published:** 2019-03-07

**Authors:** Radek Prochazka, Lucie Nemcova

**Affiliations:** Laboratory of Developmental Biology, Institute of Animal Physiology and Genetics of the Czech Academy of Sciences, Rumburska 89, 277-21 Libechov, Czech Republic; nemcova@iapg.cas.cz

**Keywords:** amphiregulin, cumulus cells, epidermal growth factor receptor, FSH, mitogen-activated protein kinase 3/1, signal transduction

## Abstract

The maturation of mammalian oocytes in vitro can be stimulated by gonadotropins (follicle-stimulating hormone, FSH) or their intrafollicular mediator, epidermal growth factor (EGF)-like peptide—amphiregulin (AREG). We have shown previously that in pig cumulus-oocyte complexes (COCs), FSH induces expression and the synthesis of AREG that binds to EGF receptor (EGFR) and activates the mitogen-activated protein kinase 3/1 (MAPK3/1) signaling pathway. However, in this study we found that FSH also caused a rapid activation of MAPK3/1 in the cumulus cells, which cannot be explained by the de novo synthesis of AREG. The rapid MAPK3/1 activation required EGFR tyrosine kinase (TK) activity, was sensitive to SRC proto-oncogene non-receptor tyrosine kinase (SRC)-family and protein kinase C (PKC) inhibitors, and was resistant to inhibitors of protein kinase A (PKA) and metalloproteinases. AREG also induced the rapid activation of MAPK3/1 in cumulus cells, but this activation was only dependent on the EGFR TK activity. We conclude that in cumulus cells, FSH induces a rapid activation of MAPK3/1 by the ligand-independent transactivation of EGFR, requiring SRC and PKC activities. This rapid activation of MAPK3/1 precedes the second mechanism participating in the generation and maintenance of active MAPK3/1—the ligand-dependent activation of EGFR depending on the synthesis of EGF-like peptides.

## 1. Introduction

MAPKs are a widely conserved family of serine/threonine protein kinases involved in many cellular processes such as cell proliferation, differentiation, motility and apoptosis. MAPKs are phosphorylated and activated by two upstream protein kinases (MAPKK and MAPKKK) that are activated by interaction with a family of small GTPases and/or other protein kinases connecting the MAPK module to cell surface receptors or external stimuli. MAPK3/1, also known as extracellular-regulated protein kinase (ERK1 and ERK2), is activated by MEK1/2 or, in vertebrate oocytes, by the protein MOS [1,2].

MAPK3/1 possesses an important role in the transduction of the signals elicited by gonadotropins in the preovulatory ovarian follicles. In mice, MAPK3/1 activity in cumulus cells is essential for gonadotropin-induced germinal vesicle breakdown (GVBD) and for the expansion of cumulus cells [3]. These conclusions are supported by the findings that activation of MAPK3/1 in cumulus cells occurred before GVBD both in vivo and in vitro, and the inhibition of MAPK3/1 activation with the MEK inhibitor blocked follicle-stimulating hormone (FSH)-induced GVBD in vitro. Moreover, it has been demonstrated that MAPK3/1 activity in cumulus cells is also required for the expression of expansion-related genes (*HAS2* and *PTGS2*), and that activation of MAPK3/1 occurs downstream of the cyclic adenosine monophosphate (cAMP) production and activation of protein kinase A (PKA) [3,4]. The transient inhibition of MAPK3/1 signaling by the i.p. injection of MEK inhibitor to equine chorionic gonadotropin/human chorionic gonadotropin (eCG/hCG)-primed mice resulted in dramatically reduced expression of genes associated with cumulus expansion and ovulation [5]. The necessity of the activation of MAPK3/1 in cumulus cells for the gonadotropin-stimulated resumption of meiosis was also demonstrated for pig cumulus-enclosed oocytes (COCs) [6,7]. Convincing evidence of the pivotal role of MAPK3/1 in the control of ovulation processes came from experiments with granulosa-cell-specific MAPK3/1 double-knockout mice that fail to ovulate and the females are completely infertile [8]. This detailed study of the ovulatory process in the the *ERK1/2^gc−/−^* mice revealed gross abnormalities in down- and upregulation of genes that are triggered by FSH or eCG during preovulatory follicle development, which indicates that MAPK3/1 is required for terminating the expression of the genes controlling the proliferation of granulosa cells as well as for inducing the genes controlling cumulus expansion, luteinization and ovulation.

The mechanisms by which MAPK3/1 regulates the preovulatory processes are not completely known, but they probably involve the activation of various transcription factors as well as a posttranscriptional modification of specific proteins in cumulus cells or oocytes. In mouse cumulus/granulosa cells, the transcription factor C/EBPβ seems to be highly affected by MAPK3/1, since disruption of the *Cebpb* gene produced a similar phenotype of granulosa cells, as reported in MAPK3/1-deficient mice [8]. The transcription factor EGR1 also belongs to potential targets of MAPK3/1, since its expression was reduced in mice with disturbed MAPK3/1 signaling, and knockdown of *EGR1* in the granulosa cell decreased the expression of *PTGS2*, a pivotal gene involved in follicular rupture [5].

The signaling pathways by which gonadotropins induce the activation of MAPK3/1 in cumulus and granulosa cells have been the subject of extensive research. The binding of gonadotropins to their specific G protein-coupled receptors on cumulus or granulosa cells leads to a release of the GTP-bound α-subunit and activation of adenylyl cyclase, generating a high concentration of cAMP in the target cells [9]. The increase in intracellular cAMP appears to be an important step in gonadotropin-induced activation of MAPK3/1, since this process can be mimicked by 8-Br-cAMP in cultured granulosa cells, which was first demonstrated by Cameron et al. [10]. In mammalian preovulatory follicles, the gonadotropin actions are mediated by expression and synthesis of epidermal growth factor (EGF)-like factors in mural granulosa cells [11,12]. These factors, amphiregulin (AREG), epiregulin (EREG) and betacellulin (BTC), bind to EGF-receptors (EGFRs), which leads to the receptor autophosphorylation and activation of several signaling pathways, including the MAPK3/1 pathway [11]. The expression of the EGF-like factors at the mRNA level increased in cultured mouse follicles about 30 min after their stimulation by LH and reached its maximum after 2 h, in perfect synchrony with EGFR phosphorylation [13]. This EGFR phosphorylation was impaired by AG1478, an EGFR kinase inhibitor, and the matrix metalloproteinase inhibitors GM6001 (galardin) and TAPI1, suggesting that shedding of EGF-like factors is required for their activation. This study further showed that the activation of MAPK3/1 was inhibited by culturing the follicles in AREG-, EREG- and BTC-neutralizing antibodies. The inhibitors AG1478 and GM6001 had only partial negative effect on MAPK3/1 activation, which suggests that other pathways are involved in this process in addition to the ligand-induced activation of the EGFR [13]. It becomes obvious that the gonadotropin-induced process of EGF-like ligand mRNA transcription, protein synthesis and shedding requires at least 1 h. However, the ability of gonadotropins to activate MAPK3/1 as fast as in 5–10 min was demonstrated by Wayne et al. [14] in cultured rat granulosa cells. Moreover, the LH-induced closure of gap junctions in cultured mouse follicles, which also depends on MAPK3/1 activity, occurred within minutes [15]. Therefore, the evidence has accumulated indicating that gonadotropins can activate MAPK3/1 by alternative pathway(s) that are quicker than the ligand-activated EGFR pathway. These alternative pathway(s) may either involve EGFR activation or skip EGFR and directly activate a signaling molecule upstream of MEK1.

The aim of this work was to monitor precisely the dynamics of MAPK3/1 activation in FSH- and AREG-stimulated pig COCs and to assess the involvement of different signaling pathways in the mechanism of MAPK3/1 activation in cumulus cells. We show here for the first time that in FSH-stimulated COCs, MAPK3/1 is rapidly activated in cumulus cells by the mechanism involving ligand-independent and SRC proto-oncogene non-receptor tyrosine kinase (SRC)- and protein kinase C (PKC)-dependent transactivation of EGFR, which is followed by the second mechanism, the ligand-dependent activation of EGFR involving the synthesis and shedding of EGF-like peptides.

## 2. Results

### 2.1. Time Course of MAPK3/1 Activation in Pig Cumulus-Enclosed Oocytes (COCs) Cultured in Medium Supplemented with Follicle-Stimulating Hormone (FSH) or Amphiregulin (AREG)

**Figure 1:**
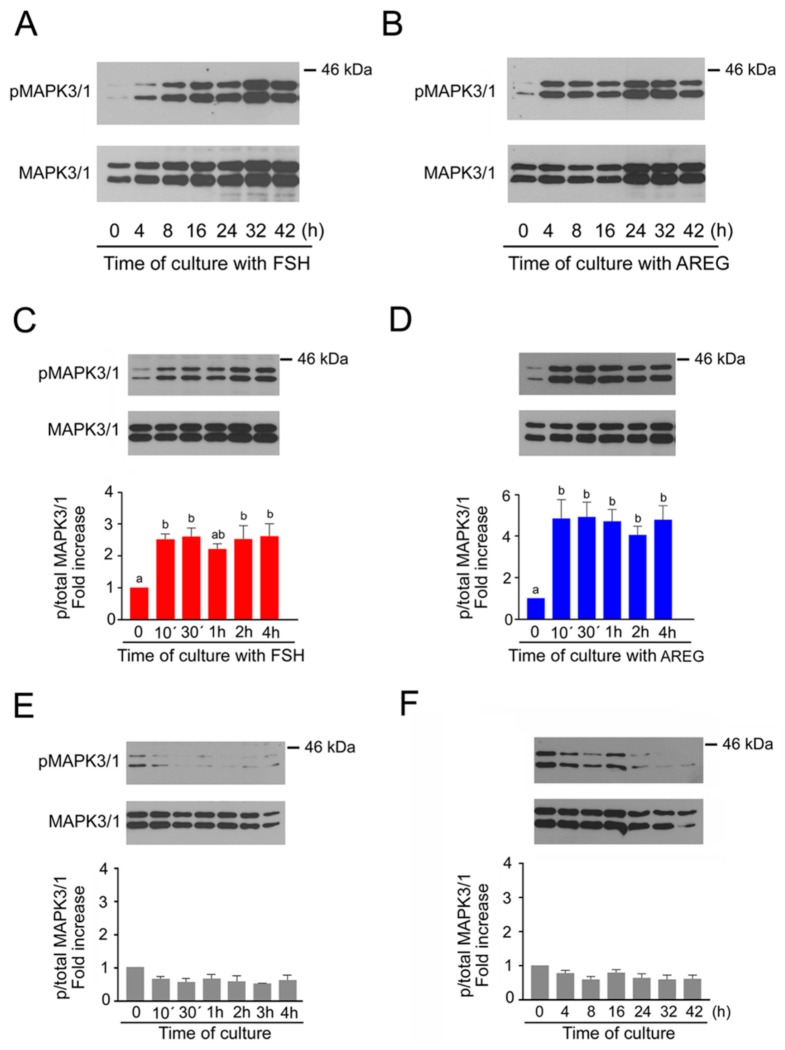
Time course of MAPK3/1 activation in cumulus-enclosed oocytes (COCs) stimulated with follicle-stimulating hormone (FSH) or amphiregulin (AREG). The panels show representative results of immunoblotting of phosphorylated and total MAPK3/1 in samples of 25 COCs cultured in vitro for the indicated periods of time. The experiments shown in panels A and B were repeated twice with the same results. The experiments shown in panels (**C**–**F**) were repeated three times. Quantification of the activated MAPK3/1 was performed by densitometry and is shown in the graphs as proportions of phosphorylated and total MAPK3/1 and expressed in arbitrary units as the fold increase over the proportion found in unstimulated COCs at the beginning of the culture. (**A**) Activation of MAPK3/1 in FSH-stimulated COCs during the long-term culture. (**B**) Activation of MAPK3/1 in AREG-stimulated COCs during the long-term culture. (**C**) Activation of MAPK3/1 in FSH-stimulated COCs during the short-term culture. (**D**) Activation of MAPK3/1 in AREG-stimulated COCs during the short-term culture. (**E**) Activity of MAPK3/1 in COCs cultured for short-term period in control medium without FSH and AREG. (**F**) Activity of MAPK3/1 in COCs cultured for long-term period in control medium without FSH and AREG. The different superscripts above the columns indicate significant differences (*p* < 0.05).

First, we assessed MAPK3/1 activity in the COCs over the whole period of culture from 0 to 42 h. As shown in Figure 1A,B, both FSH and AREG activated MAPK3/1 during the first interval of culture, i.e., within 4 h, and the activity remained high until 32 h of culture, followed by a slight decrease by the end of the culture at 42 h. In the next experiment, we focused our attention on the time course of MAPK3/1 activation during the first 4 h of culture. As shown in Figure 1C,D, a significant increase in MAPK3/1 activity over the level found in unstimulated COCs occurred within as little as 10 min after stimulation by either FSH or AREG, and the activity remained high throughout the period of culture. In contrast, the activity of MAPK3/1 did not increase in COCs cultured in the control medium without FSH or AREG (Figure 1E,F).

### 2.2. Molecular Mechanisms of FSH- and AREG-Induced Rapid Activation of MAPK3/1 in Pig COCs

**Figure 2:**
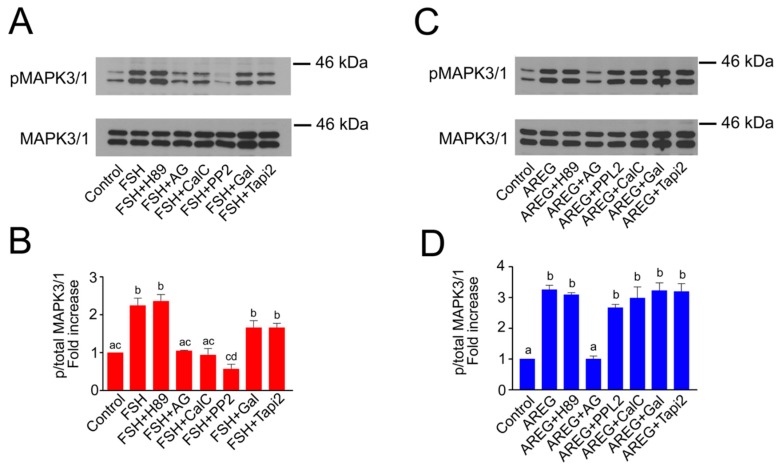
Effect of protein kinase and proteinase inhibitors on FSH- and AREG-induced rapid activation of MAPK3/1 in pig COCs. The panels show representative results of the immunoblotting of phosphorylated and total MAPK3/1 in samples of 25 COCs cultured in vitro for 10 min. The experiments were repeated three times. Quantification of the activated MAPK3/1 was performed by densitometry and is shown in the graphs as proportions of phosphorylated and total MAPK3/1, and expressed in arbitrary units as the fold increase over the proportion found in unstimulated COCs at the beginning of the culture. (**A**,**B**) show COCs stimulated by FSH; (**C**,**D**) show COCs stimulated by AREG. The different superscripts above the columns (**D**) or superscripts with no common letters (**B**) indicate significant differences (*p* < 0.05). AG: AG1478; CalC: calphostin C; Gal: galardin.

In this experiment, we assessed which kinases or metalloproteinases participate in the rapid activation of MAPK3/1 occurring in COCs within the first 10 min after stimulation. The rapid FSH-induced activation of MAPK3/1 required EGFR TK activity, since it was decreased to the basal level by AG1478. Next, it was sensitive to the SRC-family kinases inhibitor and PKC inhibitor (PP2 and calphostin C, respectively), but it was resistant to inhibitors of PKA (H89) and metalloproteinases (galardin, TAPI2) (Figure 2A,B). The phosphorylation levels of MAPK3/1 in cultures with FSH and galardin or TAPI2 were lower than in the cultures with FSH alone, but this difference was not significant. We assessed the possibility that this may be due to low concentrations of the inhibitors and carried out an experiment in which the concentration of galardin was increased from 30 to 60 and 90 µM. The results of this experiment revealed that even the highest concentration of galardin did not lower the FSH-induced rapid phosphorylation of MAPK 3/1 (Supplemental Figure S1). The rapid activation of MAPK3/1 induced by AREG was only dependent on the EGFR TK activity, i.e., it was only sensitive to AG1478, and no other inhibitor caused a significant decrease in its activity (Figure 2C,D).

### 2.3. Molecular Mechanisms Involved in Maintenance of FSH- and AREG-Induced Activation of MAPK3/1

**Figure 3:**
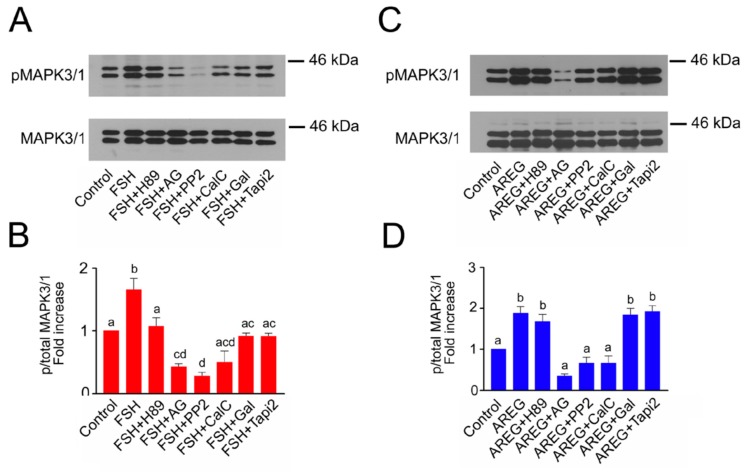
Effect of protein kinase and proteinase inhibitors on maintenance of FSH- and AREG-induced activation of MAPK3/1 in pig COCs. The panels show representative results of immunoblotting of phosphorylated and total MAPK3/1 in samples of 25 COCs cultured in vitro for 16 h. The experiments were repeated three times. Quantification of the activated MAPK3/1 was performed by densitometry and is shown in the graphs as proportions of phosphorylated and total MAPK3/1 and expressed in arbitrary units as the fold increase over the proportion found in unstimulated COCs at the beginning of the culture. (**A**,**B**) show COCs stimulated by FSH; (**C**,**D**) show COCs stimulated by AREG. The different superscripts above the columns (**D**) or superscripts with no common letters (**B**) indicate significant differences (*p* < 0.05). AG: AG1478; CalC: calphostin C; Gal: galardin.

In the next experiment, we looked at which kinases participate in the maintenance of MAPK3/1 activity in the cumulus cells for an extended period of time. For this purpose, we selected a culture interval of 16 h, i.e., before the activation of MAPK3/1 in oocytes, which occurs after more than 16 h of culture, at the time of GVBD [16]. The maintenance of the FSH-induced MAPK3/1 activity for an extended period of time was dependent on all assessed kinase- and metalloproteinase-activities (Figure 3A,B). The maintenance of AREG-induced MAPK3/1 activity was dependent on the EGFR TK, SRC and PKC activities, but not on the PKA and metalloproteinase activities (Figure 3C,D).

## 3. Discussion

The results of this study document that MAPK3/1 becomes activated in cumulus cells soon after the exposure of COCs to gonadotropins or growth factors, and that their high activity persists throughout the period of culture required for achieving full cumulus expansion and maturation of the oocyte to metaphase II. This time course of MAPK3/1 activation is in concert with the data published previously by our and other laboratories [17,18,19] and reflects the role of this kinase in the regulation of cumulus cell expansion, steroidogenesis and luteinization [20]. The onset of MAPK3/1 activation in gonadotropin-stimulated cumulus cells was recently a matter of debate, since different intervals between the exposure of COCs to gonadotropins and detection of active forms of MAPK3/1, ranging from minutes to hours, were reported [15,17,19]. The phosphorylation levels of MAPK3/1 may also be affected by culture conditions and may differ from those occurring in vivo, as documented in bovine cumulus cells [21]. It is conceivable that precise knowledge of the time course and mechanism of MAPK3/1 activation is crucial for elucidating the role of this kinase in the resumption of oocyte meiosis. The data presented in this study argue for a rapid activation of MAPK3/1 by both growth factors and gonadotropins, although the molecular mechanisms of the activation are not identical.

As expected, AREG activated MAPK3/1 after minutes, which is caused by its direct binding to EGFR, resulting in a rapid activation of the intrinsic EGFR TK, phosphorylation of the tyrosine residues in the intracellular domain of the receptor and activation of the downstream GRB2/RAS/RAF/MEK1 pathway. The speed of this signal transduction pathway, not exceeding few minutes, has been demonstrated previously [22]. We show here that the activation of MAPK3/1 by this AREG-induced pathway was only prevented by EGFR TK inhibitor, and not by PKA, PKC, SRC or metalloproteinase inhibitors. FSH also activated MAPK3/1 in 10 min. However, this activation was prevented by inhibitors of SRC and PKC, but not PKA or metalloproteinase inhibitors. These data suggest that both the FSH- and AREG-induced rapid activation of MAPK3/1 involve activation of the EGFR, but do not depend on an intracellular increase in cAMP and activation of PKA, nor on the metalloproteinase-dependent cleavage of EGF-like pro-peptides. Thus, it follows that the FSH-induced rapid activation of MAPK3/1 occurred by a ligand-independent transactivation of EGFR involving a PKC- and SRC-dependent mechanism.

The existence of the FSH-induced rapid transactivation of EGFR in follicular cells is indirectly supported by several findings. Corresponding with our data, Wayne et al. found that the FSH-induced activation of MAPK3/1 in cultured rat granulosa cells occurred in 5–10 min via a mechanism requiring SRC-family kinases and EGFR TK activities, but not PKA [14]. However, in that study, the mechanism by which SRC activates EGFR/MAPK3/1 pathway was not investigated. In addition, we show that the rapid activation of the EGFR/MAPK3/1 pathway is dependent on PKC activity, which was not studied in the paper of Wayne et al. [14]. In mice, FSH induced the translation of *TPX* and *IL7* mRNAs in the oocyte by a mechanism involving the activation of the EGFR network in cumulus cells and phosphoinositide-3 kinase/v-akt murine thymoma viral oncogene homolog (PI3K/AKT) in the oocyte [23,24]. However, the time course of oocyte AKT activation was comparable for both FSH and AREG, indicating that FSH did not activate EGFR through the stimulation of AREG synthesis, but rather via a more direct pathway. Moreover, the FSH-induced activation of AKT and translation of the reporter mRNAs worked in Areg^−/−^ mice [24]. In bovines, an SRC inhibitor (PP2) could prevent the FSH-induced gene expression pattern in cumulus cells. In addition, this pattern mirrored the effect of the EGFR inhibitor AG1478 on the FSH-induced gene expression [25]. Thus, these data indicate that both SRC and EGFR pathways are involved in the FSH-induced changes in the expression profile of bovine cumulus cells. Collectively, several papers indicated the existence of the rapid FSH-induced transactivation of EGFR in follicular cells, but the mechanism of the transactivation has not been clarified yet.

In principle, two mechanisms leading to the G-protein coupled receptor-induced activation of EGFR have been described in various cell systems [26]. First, a ligand-dependent pathway comprised of the activation of matrix metalloproteinases that are able to cleave pre-synthesized EGFR ligands such as HB-EGFR and stimulate ligand shedding. Second, a ligand-independent pathway comprised of the activation of intracellular protein tyrosine kinases such as SRC family proteins that phosphorylate EGFR in its intracellular domain. We show here for the first time that, at least in the pig model, the SRC kinase does not cause rapid activation of EGFR/MAPK3/1pathway in cumulus cells by interaction with metalloproteinases and subsequent processing of pre-existing EGF-like factors, but rather by the ligand-independent intracellular mechanism.

The SRC protein is a representative of non-receptor tyrosine kinases that are associated with transmembrane receptors, such as hormone receptors, are activated by the binding of ligands to the receptors, and work as mediators of intracellular signaling. SRC activates target proteins by the phosphorylation of their tyrosine residues. The adaptor protein GRB2 and the GTPase activator RAS belong to the targets of SRC [27] and also lie on the MAPK3/1 signaling pathway. Thus, the phosphorylation of these proteins by SRC may be implicated in the mechanisms of the FSH-induced rapid activation of MAPK3/1 in cumulus cells. However, both GRB2 and RAS lie downstream of EGFR on the MAPKs signaling pathway and our data strongly argue for the involvement of EGFR TK in this process. Thus, it appears that a SRC-mediated phosphorylation of EGFR at its cytosolic domain, described in various cell types [26,28,29,30], is the essential mechanism of the FSH-induced rapid activation of MAPK3/1 in cumulus cells. In support of this conclusion, the ligand-independent and SRC-mediated transactivation of EGFR led to activation of the MAPK3/1 pathway in different cell types [31,32].

The FSH-induced rapid activation of MAPK3/1 in pig cumulus cells was also dependent on PKC activity. This result is not surprising since the involvement of PKC in regulation of the gonadotropin-induced meiosis resumption was documented in several previous studies. The ability of pharmacological PKC activators phorbol myristate acetate (PMA) and oleoyl-acetyl-sn-glycerol (OAG) to stimulate meiotic resumption in COCs was demonstrated in mice [33,34]. A participation of MAPK3/1 in the meiosis resumption induced by PKC activators was proved by the finding that the MEK inhibitor U0126 prevented GVBD in PMA- or OAG-stimulated COCs and, vice versa, PKC inhibitors blocked FSH-induced oocyte meiotic resumption and MAPK3/1 activation [34]. In the pig COCs, PMA enhanced oocyte GVBD rate and the EGFR inhibitor AG1478 reversed this effect. The inhibition of PKC (by chelerithrine chloride) completely blocked FSH-induced meiotic resumption, but had no effect on EGF- or AREG-induced resumption, indicating a role of PKC in the FSH-mediated transactivation of EGFR [35]. Taken together, these data confirm the results of our study showing that PKC participates in the FSH-induced transactivation of EGFR.

The maintenance of MAPK3/1 activity in cumulus cells was dependent on EGFR TK, SRC and PKC activities, irrespective of whether FSH or AREG were used for its stimulation. In addition, the FSH-induced activation was further dependent on PKA and metalloproteinase activities. These data are in agreement with the previously described mechanisms of the FSH (LH)-induced activation of MAPK3/1 in cultured COCs or preovulatory follicles, which consists of the cAMP and PKA-dependent synthesis of EGF-like pro-peptides AREG, EREG and BTC, their cleavage by metalloproteinases, binding to EGFR and consequent activation of the MAPK3/1 signaling pathway [7,13]. This ligand-dependent activation of EGFR/MAPK3/1 is well supported by numerous findings. The engagement of newly synthesized proteins in LH-induced EGFR phosphorylation was evidenced by the finding that protein synthesis inhibitors violate this process as well as the resumption of oocyte meiosis [13]. This EGFR phosphorylation was inhibited by the matrix metalloproteinase inhibitors, suggesting that ligand shedding is required for their activity. The EGFR phosphorylation was mimicked in time and intensity by forskolin and inhibited by H89, indicating that cAMP and PKA signaling are upstream pathways required for the gonadotropin-induced activation of EGFR [13]. In agreement with this conclusion, the gonadotropin-induced expression of AREG, EREG and TACE/ADAM17 was also suppressed by the PKA inhibitor [36].

There are several possible ways to explain our findings that the SRC and PKC activities are also essential for the maintenance of the FSH-induced MAPK3/1 activity. First, we have shown that the FSH-induction of ligand-independent and SRC- and PKC-dependent transactivation of EGFR occurs in minutes after the onset of culture, but it is quite possible that this mechanism participates in the activation of MAPK3/1 for an extended period of time, or even throughout the period of maturation. Second, it has been shown that PKC and SRC are essential for the activation of TACE/ADAM 17 in pig cumulus and granulosa cells [37], the metalloproteinase involved in the cleavage of EGF-like pro-peptides. Third, FSH may induce other signaling pathways in cumulus cells that lead to the activation of MAPK3/1 and depend on the activity of SRC and/or PKC. For example, the binding of gonadotropins to their receptors may activate the G_i_ and G_q_ classes of the small GTP-binding proteins, which results in the activation of signal transduction kinases including PI3K and phospholipase Cβ, both of which can activate the RAS/RAF/MEK1/MAPK3/1 cascade by a mechanism involving SRC or PKC [38].

The maintenance of AREG-induced MAPK3/1 activity was, in contrast to the rapid MAPK3/1 activation, dependent on SRC and PKC. We may speculate that the long-term absence of SRC and PKC signaling may deteriorate the function of the EGFR or downstream molecules on the MAPK3/1 pathway, such as GRB2 or RAS. Interestingly, the maintenance of exogenous AREG-induced MAPK3/1 activity did not require PKA or metalloproteinase activity, which indicates that this process did not rely on the AREG-induced synthesis of EGF-like pro-peptides by the regulatory loop described previously [39] nor on the AREG/PGE2/cAMP/PKA axis described in the rodent model [40].

**Figure 4:**
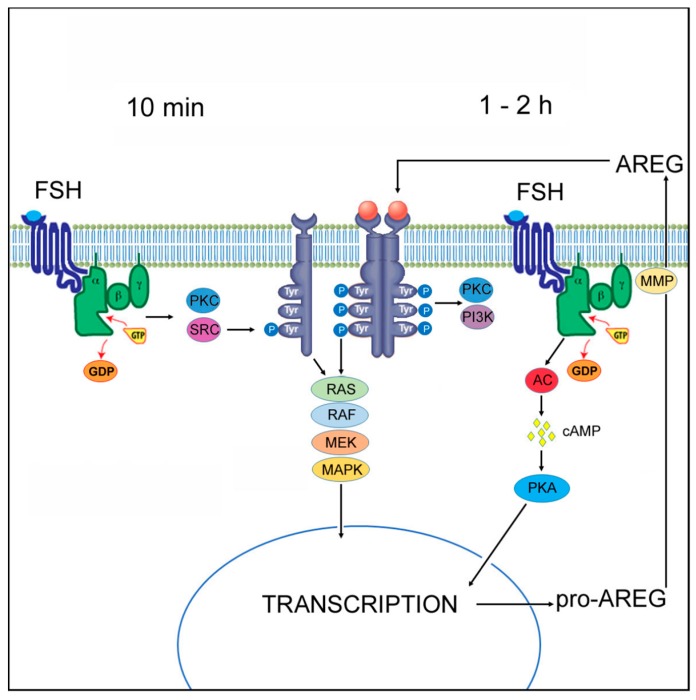
Ligand-independent and ligand-dependent transactivation of epidermal growth factor receptor (EGFR)/MAPK3/1 pathway in pig cumulus cells by FSH. The binding of FSH to the G_q_ class of G-protein coupled receptors (GPCRs) results in the activation of protein kinase C (PKC) and SRC that phosphorylates EGFR in its intracellular domain. It is not clear whether this phosphorylation leads to receptor dimerization [26]. The SRC-mediated phosphorylation is associated with activation of the RAS/RAF/MEK1 pathway and rapid activation of MAPK3/1, but it may not lead to full phosphorylation of the intracellular domain and activation of all pathways known to be associated with EGFR TK activity [31,32]. FSH also binds to the G_s_ class of GPCRs and activates adenylyl cyclase, which leads to an increase in cAMP and activation of PKA. This PKA activity is required for the synthesis of the AREG pro-peptides [13], which are cleaved at the plasma level by TACE/ADAMs metalloproteinases and released outside the cumulus cells into the extracellular space. The active forms of AREG serve as ligands for EGFR, which leads to dimerization of the receptor, full phosphorylation of its intracellular domain and activation of downstream signaling pathways, including the MAPK3/1, PKC and PI3K pathways. The transcription- and ligand synthesis-dependent process of EGFR/MAPK3/1 activation requires 1–2 h to be activated [13] and operates in cumulus cells for an extended period of time. MAPK3/1 may thus be involved in events occurring in minutes after the exposure of follicular cells to gonadotropins, such as gap junction closure through connexin phosphorylation [15] and the regulation of cGMP level in follicular cells [41,42,43], as well as in delayed events such as the regulation of steroidogenesis and cumulus expansion [20]. MEK: MEK1/2; MAPK: MAPK3/1; MMP: matrix metalloproteinases; AC: adenylyl cyclase.

In conclusion, we demonstrate here for the first time that FSH induces the rapid activation of MAPK3/1 in cumulus cells by the ligand-independent transactivation of EGFR, requiring SRC and PKC activities. This rapid activation of MAPK3/1 precedes the second mechanism participating in the generation and maintenance of active MAPK3/1—the ligand-dependent activation of EGFR depending on the de novo synthesis of EGF-like peptides (Figure 4). This finding can contribute to understanding the molecular mechanisms by which the gonadotropins induce EGFR- and MAPK3/1-dependent, minute events in follicular cells during the resumption of meiosis, such as the closure of gap junctions between granulosa/cumulus cells [15] or the decrease in cGMP production in granulosa and cumulus cells [41,42,43].

## 4. Materials and Methods

### 4.1. Culture Media and Reagents

All chemicals were purchased from Sigma (Prague, Czech Republic) unless otherwise specified.

### 4.2. Collection of Cumulus-Oocyte Complexes

Ovaries were obtained from premature crossbred gilts (Landrace and Large White), 6–8 months old and 90–120 kg in weight. The animals were slaughtered at a local abattoir, their ovaries excised and transported to the laboratory in a thermo-flask at 38 °C. The contents of medium-size antral follicles about 3–5 mm in diameter were aspirated with a syringe connected to a 20 G needle, pooled in a test-tube and allowed to sediment for 10 min. The sediment was washed twice with phosphate-buffered saline (PBS), placed in a Petri dish and the COCs were collected with a pipette. Only COCs surrounded by a compact multi-layered cumulus were selected for experiments.

### 4.3. Culture of Cumulus-Oocyte Complexes In Vitro

The COCs were cultured in M-199 medium (Gibco, Life Technologies, Rockville, MD, USA) supplemented with 0.91 mM sodium pyruvate, 0.57 mM cysteine, 5.5 mM Hepes, antibiotics and fetal calf serum (5%). Groups of 25–30 COCs were cultured in four-well dishes (Nunclon, Roskilde, Denmark) in 0.5 mL of culture medium at 38.5°C in a humidified atmosphere of 5% CO_2_. To stimulate the expansion of cumulus cells and oocyte maturation, the culture medium was supplemented with 1 IU/mL of human recombinant FSH (Gonal-f, Merck Serono, London, UK) or 100 ng/mL of AREG. The following inhibitors were added to the culture medium with the aim of assessing their effect on MAPK3/1 activation: H89, a PKA inhibitor (20 µM); AG1478, an EGFR tyrosine kinase inhibitor (5 µM); calphostin C, a PKC inhibitor (5 µM); PP2, a SRC kinase inhibitor (Merck, Darmstadt, Germany; 10 µM); galardin and TAPI2, metalloproteinase inhibitors (Merck; 30 and 50 µM, respectively). The inhibitors, except for TAPI2, were dissolved in dimethyl sulfoxide (DMSO) and 10 mM stocks were stored frozen at −20 °C for a maximum period of 3 months. TAPI2 was dissolved in PBS. The COCs were first exposed to the inhibitors for 1 h and then FSH or AREG were added to culture wells. The concentrations of the inhibitors were selected on the basis of the data published previously by our [7] and other laboratories. The maximum concentration of DMSO in culture wells with added inhibitors was 0.3 % (v/w); this concentration of DMSO was also used in the control and FSH-supplemented media.

### 4.4. Immunoblotting

At the selected culture time, COCs were washed in PBS and solubilized in Laemmli buffer containing 2% sodium dodecyl sulphate (SDS) and 5% 2-mercaptoethanol. Samples were boiled at 100°C for 3 min and stored frozen at −20 °C. Subsequently, proteins were separated in 10% acrylamide/SDS gels and transferred to Immobilon-P membranes (Millipore, Bedford, MA, USA). Membranes were blocked in 5% low-fat dry milk in Tris-buffered saline (TBS) with 0.5% Tween 20 for 2 h at room temperature and then incubated with a primary antibody diluted 1:1000 in 5% BSA in TBS-Tween, at 4 °C overnight. The primary antibodies were p-ERK and ERK1 (detecting MAPK3/1), both from Santa Cruz Biotechnology (Santa Cruz, CA, USA). The secondary antibodies (Amersham ECL anti-mouse or anti-rabbit IgG, GE Healthcare, Little Chalfont, UK) were diluted 1:5000 in 2% BSA in TBS-Tween. The membranes were incubated with the secondary antibody for 1 h at room temperature and washed intensively in TBS-Tween. The immune reaction was detected by enhanced chemiluminescence (Pierce, Rockford, IL, USA) according to the manufacturer’s instructions. The intensity of the specific bands on the blots was analyzed by scanning densitometry using the free software Image J Version 1.29 (National Institute of Mental Health, Bethesda MD, USA).

### 4.5. Statistical Analysis

The statistical analyses were performed with the software GraphPad Prism 5.0 (La Jolla, CA, USA). Each experiment was performed in at least 3 replicates. The densitometrical quantifications of MAPK3/1 were compared by analysis of variance (ANOVA) followed by Tukey’s post-test. A level of *p* < 0.05 was considered significant. Error bars indicate the standard error of the mean (SEM).

## Supplementary Materials

The supplementary materials are available online at https://www.mdpi.com/1422-0067/20/5/1179/s1. Figure S1. Effect of metalloproteinase inhibition by different concentration of galardin on FSH-induced rapid activation of MAPK3/1 in pig cumulus-oocyte complexes. (**A**) The panel shows representative results of immunoblotting of phosphorylated and total MAPK3/1 in samples of 25 COCs cultured in vitro for 10 min. (**B**) Quantification of the activated MAPK3/1 was performed by densitometry and is shown in the graph as proportions of phosphorylated and total MAPK3/1 and expressed in arbitrary units as fold increase over the proportion found in unstimulated COCs at the beginning of the culture. The experiments were repeated three times. C: control; F: FSH; Gal: galarding.

## References

[1] Posada J., Yew N., Ahn N.G., Vande Woude G.F., Cooper J.A. (1993). Mos stimulates MAP kinase in Xenopus oocytes and activates a MAP kinase kinase in vitro. Mol. Cell. Biol..

[2] Verlhac M.H., Lefebvre C., Kubiak J.Z., Umbhauer M., Rassinier P., Colledge W., Maro B. (2000). Mos activates MAP kinase in mouse oocytes through two opposite pathways. EMBO J..

[3] Su Y., Wigglesworth K., Pendola F.L., O’Brien M.J., Eppig J.J. (2002). Mitogen-activated protein kinase activity in cumulus cells is essential for gonadotropin-induced oocyte meiotic resumption and cumulus expansion in the mouse. Endocrinology.

[4] Su Y.Q., Denegre J.M., Wigglesworth K., Pendola F.L., O’Brien M.J., Eppig J.J. (2003). Oocyte-dependent activation of mitogen-activated protein kinase (ERK1/2) in cumulus cells is required for the maturation of the mouse oocyte-cumulus cell complex. Dev. Biol..

[5] Siddappa D., Beaulieu É., Gévry N., Roux P.P., Bordignon V., Duggavathi R. (2015). Effect of the transient pharmacological inhibition of Mapk3/1 pathway on ovulation in mice. PLoS ONE.

[6] Meinecke B., Krischek C. (2003). MAPK/ERK kinase (MEK) signalling is required for resumption of meiosis in cultured cumulus-enclosed pig oocytes. Zygote.

[7] Prochazka R., Blaha M., Nemcova L. (2012). Signaling pathways regulating FSH- and amphiregulin-induced meiotic resumption and cumulus cell expansion in the pig. Reproduction.

[8] Fan H.Y., Liu Z., Shimada M., Sterneck E., Johnson P.F., Hedrick S.M., Richards J.S. (2009). MAPK3/1 (ERK1/2) in ovarian granulosa cells are essential for female fertility. Science.

[9] Mehlmann L.M. (2005). Stops and starts in mammalian oocytes: Recent advances in understanding the regulation of meiotic arrest and oocyte maturation. Reproduction.

[10] Cameron M.R., Foster J.S., Bukovsky A., Wimalasena J. (1996). Activation of mitogen-activated protein kinases by gonadotropins and cyclic adenosine 5′-monophosphates in porcine granulosa cells. Biol. Reprod..

[11] Park J.Y., Su Y.Q., Ariga M., Law E., Jin S.L., Conti M. (2004). EGF-like growth factors as mediators of LH action in the ovulatory follicle. Science.

[12] Ashkenazi H., Cao X., Motola S., Popliker M., Conti M., Tsafriri A. (2005). Epidermal growth factor family members: Endogenous mediators of the ovulatory response. Endocrinology.

[13] Panigone S., Hsieh M., Fu M., Persani L., Conti M. (2008). Luteinizing hormone signaling in preovulatory follicles involves early activation of the epidermal growth factor receptor pathway. Mol. Endocrinol..

[14] Wayne C.M., Fan H.Y., Cheng X., Richards J.S. (2007). Follicle-stimulating hormone induces multiple signaling cascades: Evidence that activation of Rous sarcoma oncogene, RAS, and the epidermal growth factor receptor are critical for granulosa cell differentiation. Mol. Endocrinol..

[15] Sela-Abramovich S., Chorev E., Galiani D., Dekel N. (2005). Mitogen-activated protein kinase mediates luteinizing hormone-induced breakdown of communication and oocyte maturation in rat ovarian follicles. Endocrinology.

[16] Ohashi S., Naito K., Sugiura K., Iwamori N., Goto S., Naruoka H., Tojo H. (2003). Analyses of mitogen-activated protein kinase function in the maturation of porcine oocytes. Biol. Reprod..

[17] Ebeling S., Schuon C., Meinecke B. (2007). Mitogen-activated protein kinase phosphorylation patterns in pig oocytes and cumulus cells during gonadotrophin-induced resumption of meiosis in vitro. Zygote.

[18] Nemcova L., Nagyova E., Petlach M., Tomanek M., Prochazka R. (2007). Molecular mechanisms of insulin-like growth factor 1 promoted synthesis and retention of hyaluronic acid in porcine oocyte-cumulus complexes. Biol. Reprod..

[19] Yamashita Y., Okamoto M., Kawashima I., Okazaki T., Nishimura R., Gunji Y., Hishinuma M., Shimada M. (2011). Positive feedback loop between prostaglandin E2 and EGF-like factors is essential for sustainable activation of MAPK3/1 in cumulus cells during in vitro maturation of porcine cumulus oocyte complexes. Biol. Reprod..

[20] Prochazka R., Blaha M. (2015). Regulation of mitogen-activated protein kinase 3/1 activity during meiosis resumption in mammals. J. Reprod. Dev..

[21] Salhab M., Dhorne-Pollet S., Auclair S., Guyader-Joly C., Brisard D., Dalbies-Tran R., Dupont J., Ponsart C., Mermillod P., Uzbekova S. (2013). In vitro maturation of oocytes alters gene expression and signaling pathways in bovine cumulus cells. Mol. Reprod. Dev..

[22] Reddy R.J., Gajadhar A.S., Swenson E.J., Rothenberg D.A., Curran T.G., White F.M. (2016). Early signaling dynamics of the epidermal growth factor receptor. Proc. Natl. Acad. Sci. USA.

[23] Chen J., Torcia S., Xie F., Lin C.J., Cakmak H., Franciosi F., Horner K., Onodera C., Song J.S., Cedars M.I. (2013). Somatic cells regulate maternal mRNA translation and developmental competence of mouse oocytes. Nat. Cell Biol..

[24] Franciosi F., Manandhar S., Conti M. (2016). FSH regulates mRNA translation in mouse oocytes and promotes developmental competence. Endocrinology.

[25] Khan D.R., Guillemette C., Sirard M.A., Richard F.J. (2015). Characterization of FSH signalling networks in bovine cumulus cells: A perspective on oocyte competence acquisition. Mol. Hum. Reprod..

[26] Wang Z. (2016). Transactivation of Epidermal Growth Factor Receptor by G Protein-Coupled Receptors: Recent Progress, Challenges and Future Research. Int. J. Mol. Sci..

[27] Tatosyan A.G., Mizenina O.A. (2000). Kinases of the Src family: Structure and functions. Biochemistry.

[28] Liebmann C. (2011). EGF receptor activation by GPCRs: An universal pathway reveals different versions. Mol. Cell. Endocrinol..

[29] George A.J., Hannan R.D., Thomas W.G. (2013). Unravelling the molecular complexity of GPCR-mediated EGFR transactivation using functional genomics approaches. FEBS J..

[30] Cattaneo F., Guerra G., Parisi M., De Marinis M., Tafuri D., Cinelli M., Ammendola R. (2014). Cell-surface receptors transactivation mediated by G protein-coupled receptors. Int. J. Mol. Sci..

[31] Drube S., Stirnweiss J., Valkova C., Liebmann C. (2006). Ligand-independent and EGF receptor-supported transactivation: Lessons from beta2-adrenergic receptor signalling. Cell Signal..

[32] Chen Y., Peng F.F., Jin J., Chen H.M., Yu H., Zhang B.F. (2017). Src-mediated ligand release-independent EGFR transactivation involves TGF-β-induced Smad3 activation in mesangial cells. Biochem. Biophys. Res. Commun..

[33] Downs S.M., Cottom J., Hunzicker-Dunn M. (2001). Protein kinase C and meiotic regulation in isolated mouse oocytes. Mol. Reprod. Dev..

[34] Fan H.Y., Huo L.J., Chen D.Y., Schatten H., Sun Q.Y. (2004). Protein kinase C and mitogen-activated protein kinase cascade in mouse cumulus cells: Cross talk and effect on meiotic resumption of oocyte. Biol. Reprod..

[35] Chen X., Zhou B., Yan J., Xu B., Tai P., Li J., Peng S., Zhang M., Xia G. (2008). Epidermal growth factor receptor activation by protein kinase C is necessary for FSH-induced meiotic resumption in porcine cumulus-oocyte complexes. J. Endocrinol..

[36] Yamashita Y., Hishinuma M., Shimada M. (2009). Activation of PKA, p38 MAPK and ERK1/2 by gonadotropins in cumulus cells is critical for induction of EGF-like factor and TACE/ADAM17 gene expression during in vitro maturation of porcine COCs. J. Ovarian Res..

[37] Yamashita Y., Okamoto M., Ikeda M., Okamoto A., Sakai M., Gunji Y., Nishimura R., Hishinuma M., Shimada M. (2014). Protein kinase C (PKC) increases TACE/ADAM17 enzyme activity in porcine ovarian somatic cells, which is essential for granulosa cell luteinization and oocyte maturation. Endocrinology.

[38] Goldsmith Z.G., Dhanasekaran D.N. (2007). G protein regulation of MAPK networks. Oncogene.

[39] Shimada M., Hernandez-Gonzalez I., Gonzalez-Robayna I., Richards J.S. (2006). Paracrine and autocrine regulation of epidermal growth factor-like factors in cumulus oocyte complexes and granulosa cells: Key roles for prostaglandin synthase 2 and progesterone receptor. Mol. Endocrinol..

[40] Gilchrist R.B., Luciano A.M., Richani D., Zeng H.T., Wang X., Vos M.D., Sugimura S., Smitz J., Richard F.J., Thompson J.G. (2016). Oocyte maturation and quality: Role of cyclic nucleotides. Reproduction.

[41] Egbert J.R., Shuhaibar L.C., Edmund A.B., Van Helden D.A., Robinson J.W., Uliasz T.F., Baena V., Geerts A., Wunder F., Potter L.R. (2014). Dephosphorylation and inactivation of NPR2 guanylyl cyclase in granulosa cells contributes to the LH-induced decrease in cGMP that causes resumption of meiosis in rat oocytes. Development.

[42] Egbert J.R., Uliasz T.F., Shuhaibar L.C., Geerts A., Wunder F., Kleiman R.J., Humphrey J.M., Lampe P.D., Artemyev N.O., Rybalkin S.D. (2016). Luteinizing hormone causes phosphorylation and activation of the cGMP phosphodiesterase PDE5 in rat ovarian follicles, contributing, together with PDE1 activity, to the resumption of meiosis. Biol. Reprod..

[43] Shuhaibar L.C., Egbert J.R., Edmund A.B., Uliasz T.F., Dickey D.M., Yee S.P., Potter L.R., Jaffe L.A. (2016). Dephosphorylation of juxtamembrane serines and threonines of the NPR2 guanylyl cyclase is required for rapid resumption of oocyte meiosis in response to luteinizing hormone. Dev. Biol..

