# Supplementary material for: Mechanisms of FSH- and Amphiregulin-Induced MAP Kinase 3/1 Activation in Pig Cumulus-Oocyte Complexes During Maturation In Vitro
Source: Int J Mol Sci. 2019 Mar 7;20(5):1179. doi: 10.3390/ijms20051179 (PMC6429514; doi:10.3390/ijms20051179)
Supplement: Supplementary file 1 [file ijms-20-01179-s001.pdf]

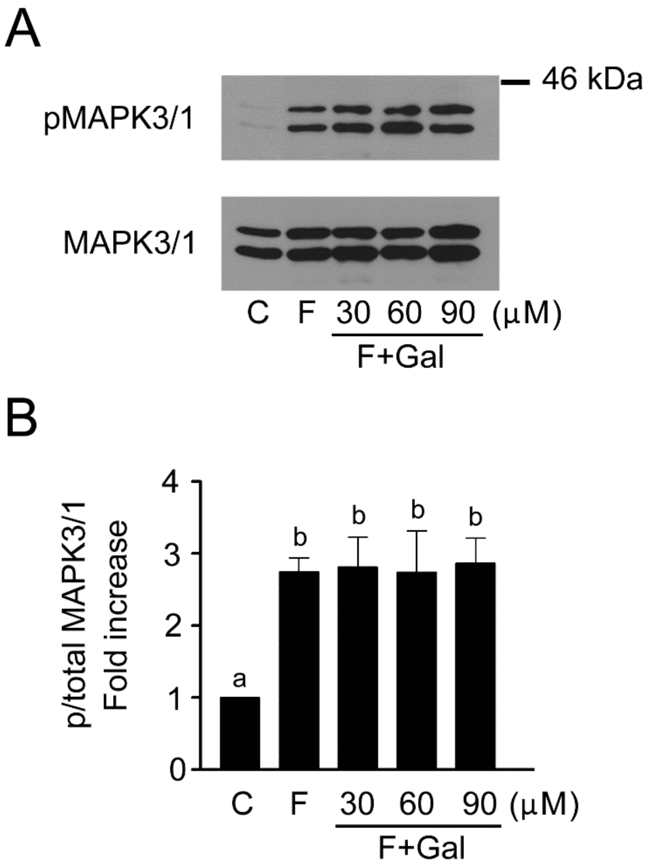

Figure S1. Effect of metalloproteinase inhibition by different concentration of galardin on FSH-induced rapid activation of MAPK3/1 in pig cumulus-oocyte complexes.
